# Current News and Opinion

**Published:** 1885-06

**Authors:** 


### AMERICAN DENTAL ASSOCIATION.

The next meeting of this society will be held in Minneapolis, the first week in August. Every member should now begin to prepare for it.

### Current News and Opinion.

### FOURTEENTH ANNUAL MEETING OF THE KANSAS STATE DENTAL ASSOCIATION.

The Kansas State Dental Society met at Topeka, May 5th, and held a meeting of great profit and interest. Papers were read by Drs. L. C. Wasson,, J. D. Patterson, R. I. Pearson, and others. During the meeting, Dr. Patterson, on behalf of the society, presented Dr. L. C. Wasson, who is one of the State Senators of Kansas, with a beautiful gold headed cane, in recognition of his successful efforts for the passage of the State Dental Law. As soon as Dr. Wasson had closed his feeling response, Dr. Thompson, of Topeka, rose and presented Dr. R. I. Pearson with another gold headed cane, in recognition of his services in the same cause, and for the great interest he had manifested in the State Society. Both gentlemen were completely surprised, but managed to express their pleasure in this mark of the esteem of their brethren.

The following resolutions were unanimously adopted :

WHEREAS. Since the last meeting of the Kansas State Dental Association, the all-wise author of our being has removed by death, our highly esteemed friend and fellow-laborer, Dr. L. P. Meredith. Therefore

*Resolved*, That we desire to give expression to our sincere regret that one so valuable to the dental profession as an author and as a cultivated gentleman, and one of such high professional attainments should be lost to the cause of dental progress in the world.

*Resolved*, That we will profit by his counsels, which were always so ably given.

*Resolved*, That a copy of these resolutions be spread upon a memorial page in the association records, a copy furnished to the family of the deceased, and to the dental journals.

*Resolved*, That we extend to his sorrowing family our sincere sympathy in their deep affliction.

J. D. PATTERSON,<br>R. I. PEARSON,<br>A. H. THOMPSON. } *Committee.*

C. B. REED, Secretary.

### KENTUCKY STATE DENTAL ASSOCIATION.

The fifteenth annual meeting of this society will be held at the Galt House, Louisville, commencing Tuesday, June 2, 1885, at 2 o'clock, P. M. The executive committee have prepared an excellent programme, and a full attendance is expected. All practicing dentists are invited to be present.

R. C. MORGAN, Pres'dt, Lancaster.

C. E. DUNN, Sec'y, Louisville.

### DENTAL LAW IN MINNESOTA.

Pursuant to a law entitled "An Act to regulate the practice of Dentistry in the State of Minnesota," approved March 3, 1885, the Governor of the State has appointed as the first Board of Dental Examiners the following practitioners:

J. I. Clements, D. D. S., *President*, Faribault.

G. V. I. Brown, D. D. S., St. Paul.

G. B. Merry, D. D. S., Stillwater.

M. R. Metcalf, D. D. S., Duluth.

J. H. Martindale, M. D., D. D. S., *Secretary*, Minneapolis.

The board is required by the law, first, to demand of all dentists who were practicing in the State of Minnesota previous to March 3, 1885, that they register with them in a book kept for the purpose; secondly, to require of all dentists who shall have come to the State after said date, that they pass a satisfactory examination in the science and art of dental surgery, or present diplomas from reputable dental colleges.

The members of the board at their first meeting unanimously agreed to execute, so far as in them lies, the full spirit and letter of the law, and to work in harmony with the National Board of Dental Examiners, as far as possible.

J. H. MARTINDALE, Secretary.

---

### MISSOURI STATE DENTAL ASSOCIATION.

The twenty-first annual meeting of the Missouri State Dental Association will be held at Sweet Springs, Mo., commencing July 7, 1885. All are cordially invited to visit this beautiful watering place, and attend this meeting.

By order of exec. committee,
J. D. PATTERSON,
C. B. HEWITT,
E. E. SHATTUCK.

---

### THE WOODEN TOOTHPICK.

A traveling man called on me yesterday morning, with his face badly swollen. He said he had visited an operator in a western city, and was told that he had an "ulcerated tooth." Upon examination I found a high state of inflammation, and pus exuding freely from between the first and second right superior molars. The patient stated that this state of things had been going on for two weeks, with no relief. The teeth were perfectly sound. While using the probe, I detected a foreign substance; on removing it I found it to be a piece of wooden toothpick, half an inch long. On seeing it he remarked, "I broke that off six weeks ago, but thought I had it all out." This is the second case of the kind that I have had in less than six months. It is about time that wooden toothpicks were discarded. RATHBUN.

---

NATIONAL ASSOCIATION OF DENTAL FACULTIES.

The annual meeting of the National Association of Dental Faculties will be held at the Sherman House, in Chicago, at 11 o'clock, A. M., July 31st. By order of the executive committee.

H. A. SMITH, Secretary. C. N. PIERCE. President.

ARTICLE VII OF THE CONSTITUTION.

*Membership:* Any reputable dental college may be represented in this body upon submitting to the executive committee satisfactory credentials, signing the constitution, conforming to the rules and regulations of this body, and paying such assessments as may be made.

---

AMERICAN DENTAL SOCIETY OF EUROPE.

The thirteenth annual meeting of the American Dental Society of Europe will be held in Berlin, on August 25, 26, and 27, 1885. Members having communications to present are requested to notify the recording secretary of the same at an early date. It is the duty of every member to work for the standing of the Society and of American Dentistry in Europe. "No time" is closely related to no inclination.

OFFICERS:

*President*, W. D. Miller, Berlin; *Vice-President*, H. C. Edwards, Madrid; *Treasurer*, E. De Trey, Vevey; *Secretary*, F. Foerster, Berlin.

---

CONNECTICUT VALLEY AND MASSACHUSETTS DENTAL SOCIETIES.

The Connecticut Valley Dental Society, and the Massachusetts Dental Society, will hold a joint convention at Worcester, Wednesday and Thursday, June 24th and 25th.

A number of the prominent members of each society will present papers, and the executive committees have arranged to make this meeting unusually interesting and profitable.

A cordial invitation is extended to all members of the profession.

The Bay State House will be the head-quarters of both societies.

GEO. A. MAXFIELD, Secretary.

(58.) Three weeks ago I placed in the mouth of a patient, a lady of middle age, a full set of teeth on ordinary red rubber. The pieces were well vulcanized, but almost immediately a profuse flow of saliva commenced, which has continued ever since—about three weeks. They do not hurt her, and she can use them comfortably, but while she is speaking the saliva will flow out of her mouth, almost in a stream. Undoubtedly, some dentists will insist that it is the mercury in the rubber, but I have never observed any such ill effects before, and I have made a great many such plates. Cannot some one enlighten me upon the subject. OLD DENTIST.

(59.) Mothers frequently apply to me, bringing young children with abscessed temporary teeth. I fully recognize the fact that the first teeth should not be extracted, if it is possible to keep them in. But we cannot leave the little ones to suffer continually, and some relief must be given. Extraction is a painful process, and inspires the little ones with such a horror of the dentist that it is almost impossible ever to get them inside the office again. It is not practicable to fill the roots, as we might in adult teeth. I should be glad to know the practice of dentists generally in these cases, and will be obliged if some one having an extended experience will answer. B. C. M.

(60) In the April number of this journal is an editorial, in which we are told that cocaine acts only upon nervous tissue. I should like to be informed *how* it acts, and in what manner it suspends sensation? P. W. B.

(61.) I have not seen or heard much lately concerning the bleaching of teeth that were discolored. Has this practice been abandoned? I have a bad case, and would be glad of advice as to how best to proceed? DENTOS.

(62.) I have lately been using Peroxide of Hydrogen in the treatment of chronic abscess, and it works like a charm. I see that it is recommended by Dr. Harlan, that after an application of this it is best to succeed it with another remedy. Will he tell us why, so long as this seems to do the work? BYRON.

(63.) Will some reader of the INDEPENDENT PRACTITIONER kindly favor me with the formula for making "Liquid Silex?" R. C. M., Germany.

---

### Answers.

R. (53.) Use dialysed iron to neutralize the arsenious acid that may have escaped from the cavity when devitalizing a pulp. B.

P. W. B. (60.) It is impossible, within the limits of this page, to present a treatise upon the physiological effects of any drug. It is sufficient to say that to paralyze nervous tissue is the specific office of anæsthetics. It is in this way that chloroform acts. It is carried by the blood to the arterioles, where it meets the terminal nerve filaments, and its action is to suspend their functions and to interrupt nervous currents. The primary effect is at the periphery of the nerves, and the progress of anæsthesia is toward the great ganglia. Cocaine will not produce its characteristic effects unless placed in actual contact with nerve tissue. Its action is lost if it be introduced into the blood. The primary action of strychnia, woorari, hydrocyanic acid, etc., is directly the opposite to that of chloroform, ether, and nitrous oxide, as their primary action is on the nerve centres, the influence extending towards the periphery. The action of all these drugs is *specific*, and not through nutritive, or (probably) molecular changes. EDITOR.

We are always glad to receive communications for this, the *practical* department of the INDEPENDENT PRACTITIONER. Let those who desire information ask it, and those who have knowledge impart it.—*Editor*.

